# Investigation of biofilm production and its association with genetic and phenotypic characteristics of OM (osteomyelitis) and non-OM orthopedic *Staphylococcus aureus*

**DOI:** 10.1186/s12941-020-00352-4

**Published:** 2020-03-26

**Authors:** Shengpeng Yu, Bei Jiang, Chao Jia, Hongri Wu, Jie Shen, Xiaomei Hu, Zhao Xie

**Affiliations:** 1 Department of Orthopedics, Southwest Hospital, Army Medical University, Gaotanyan Main Street 30#, District Shapingba, Chongqing, China; 2 Department of Orthopedics, Dujiangyan Medical Center, Dujiangyan, Sichuan China; 3 Department of Microbiology, College of Basic Medical Sciences, Army Medical University, Gaotanyan Main Street 30#, District Shapingba, Chongqing, China

**Keywords:** *Staphylococcus aureus*, Biofilm, Orthopedic infection, Osteomyelitis

## Abstract

**Background:**

*Staphylococcus aureus* is a primary pathogen of orthopedic infections. By mediating antimicrobial resistance, *S. aureus* biofilm plays an important role in the recalcitrance of orthopedic infections, especially for the intractable osteomyelitis (OM). This study investigated the relationship between biofilm production and various genetic or phenotypic characteristics among orthopedic *S. aureus* strains.

**Methods:**

A total of 137 orthopedic *S. aureus* isolates were enrolled and divided into OM and non-OM groups. Biofilm production was evaluated using the crystal violet assay. Genetic and phenotypic characteristics including MRSA identification, MLST and *spa* typing, carriage of virulence genes, drug resistance, and patients’ inflammatory responses indicators were characterized. The relationship between biofilm production and above-mentioned features was respectively analyzed among all isolates and compared between OM and non-OM isolates.

**Results:**

Biofilm production presented no significant difference between OM (including 9 MRSA isolates) and non-OM (including 21 MRSA isolates) strains. We found that ST88, t377 and ST630-MSSA-t377 strains produced very strong biofilms, while MLST types of ST15, ST25, ST398, ST5, ST59 and *spa* types of t002, t2325, t437 tended to produce weaker biofilms. Strains with the following profiles produced stronger biofilms: *fib*(+)-*hlgv*(+)-*lukED*(+)-*sei*(-)-*sem*(-)-*seo*(-) for all isolates, *sei*(-)-*sem*(-)-*seo*(-) for OM isolates, and *cna* (+)-*fib* (+)-*hlgv* (+)-*lukED* (+)-*seb*(-)-*sed*(-) for non-OM isolates. In addition, not any single drug resistance was found to be related to biofilm production. We also observed that, among OM patients, strains with stronger biofilms caused weaker inflammatory responses.

**Conclusion:**

Some genetic or phenotypic characteristics of orthopedic strains were associated with biofilm production, and this association could be different among OM and non-OM strains. The results are of great significance for better understanding, evaluating and managing different kinds of biofilm-associated orthopedic infections, and provide potential targets for biofilm clearance.

## Introduction

*Staphylococcus aureus* is a common and versatile gram-positive pathogen in orthopedic patients [1, 2]. The notorious bacterium attracts more and more attention because of its ability of producing various virulence factors and the worsening situation of drug resistance [3]. Biofilm is an important tool of pathogenic bacteria, and composed of bacterial communities and the polymeric matrix produced by them [4, 5]. Biofilm-associated infections are challenging for anti-infectious therapy, because the biofilm structure provides an ideal shelter for the bacteria to survive from antimicrobial killing and clearance of immune system [5, 6]. It was reported that biofilm bacteria could be 10 to 1000 times more resistant than the planktonic bacteria [7].

*Staphylococcus aureus* biofilm infections are very common in orthopedic infections [2, 5, 8]. It has been widely recognized that this situation not only makes the bacteria difficult to eradicate, but also makes the infections tend to deteriorate into chronic and recurrent courses [6, 8]. Osteomyelitis (OM) is one of the most serious orthopedic infectious diseases, bringing huge physical and economic burdens to the patients [9]. The problem of *S. aureus* biofilm becomes more intractable when a patient suffers from OM infection [8, 10]. Although the contribution of biofilm to *S. aureus* pathogenicity has been widely studied, few studies focused on exploring the relationship between biofilm production and genetic or phenotypic characteristics of orthopedic *S. aureus* isolates, especially for *S. aureus* OM isolates.

In a previous study conducted by our team [9], we collected and characterized the *S. aureus* isolates obtained from the orthopedic center of our hospital over a 2-year period. In consideration that genetic and phenotypic characteristics of bacteria strains are closely related to the biological and physiological properties [6, 8, 11–13], we hypothesized that some of these characteristics might be associated with biofilm formation, and the association might be different between OM and non-OM isolates. Therefore, interested isolates from the previous study were included and divided into OM and non-OM isolates, biofilm production was measured using the crystal violet assay. The relationship between biofilm production and various genetic and phenotypic features (including the molecular type, carriage of the specific virulence gene, drug resistance, and inflammatory responses of corresponding patients) of the bacterial strains were investigated. Results of the study will reveal the genetic and phenotypic features of the *S. aureus* OM and non-OM strains with different capacities of biofilm production, and provide potential indicators for strong biofilm producers to help clinicians get early attention to the difficult-to-treat infections.

## Methods

### Bacterial isolates

This study was conducted at the orthopedic center of Southwest Hospital (First Affiliated Hospital of Army Military Medical University), a tertiary hospital in southwest China. All the available orthopedic *S. aureus* infection strains (162 isolates) isolated from inpatients of our center admitted from September 2013 to September 2015 were obtained [9]. All isolates were identified as *S. aureus* by phenotypic methods (API staphy system, Biomerieux), and further confirmed by PCR of the *S. aureus*-specific gene *femB* [9, 14]. For the same patient, only the first positive culture was enrolled (23 isolates were excluded). Two isolates were excluded because of the missing of basic information. Finally, a total of 137 *S. aureus* isolates were included in this study. Included strains were divided into OM group (isolating from the marrow or its adjacent tissues of *S. aureus* OM infection, 60 isolates) and non-OM group (isolating from contemporaneous inpatients that had never been diagnosed as OM, specimen sources include wounds, tissues, joint fluid, pus and blood, 77 isolates) [9].

### Definitions

MRSA (methicillin-resistant *Staphylococcus aureus*) was identified when the *mecA* gene was positive on the genome of a *S. aureus* isolate by PCR method. An isolate was considered multi-drug resistance (MDR) when it was resistant to three or more classes of non-β-lactam antimicrobials. A clonal complex (CC) was defined to contain at least two STs sharing any six of the seven alleles [14]. *S. aureus* OM was diagnosed based on clinical symptoms, microbiology, histopathology, laboratory studies, and imaging examinations [15].

### Biofilm production assay

Biofilm production was measured using the crystal violet assay. TSB (tryptic soy broth) medium supplemented with 1% glucose and 2% NaCl was used for cultivation of biofilm production [16, 17]. Overnight culture of each isolate was respectively diluted 1:100 into 200 μl medium in a 96-well flat-bottom plate (Costar-3599, USA). After incubation at 37 °C for 24 h, the supernatants were removed, and the wells were washed three times with PBS. Biofilm was fixed in an incubator at 60 °C for 15 min, and then 150 μl of 0.1% crystal violet solution was added to each well containing dry biofilm. After 15 min of staining, the plate was rinsed with PBS and air dried. Subsequently, 150 μl of 33% acetic acid was added to each well to resuspend the stained biofilm, and the optical density at 492 nm (OD_492_) was measured using a Multiskan GO microplate reader (Thermo Scientific, USA). The well containing only sterile medium without bacteria inoculation was used as a negative control. A good biofilm former *S. aureus* ATCC 29213 was used as a positive control. Each isolate was tested for at least three biological repeats.

The production of biofilm formation was assessed by the OD_492_ value and multiples of cut-off OD value (ODc). ODc was calculated from arithmetic mean of the OD_492_ of negative controls with three times addition of standard deviation. Specifically, for each biological repeat, three negative control wells in each 96-well plate were designed (two 96-well plates were used in each biological repeat), the arithmetic mean of negative controls was then calculated. In this way, three arithmetic means could be acquired by three biological repeats, and then they were used for calculating the final arithmetic mean and standard deviation to get the ODc value. The following classification was applied to determine the capacity of biofilm formation: no biofilm production (OD ≤ ODc), weak bioflim production (ODc < OD ≤ 2ODc), moderate biofilm production (2ODc < OD ≤ 4ODc), and strong biofilm production (OD > 4ODc) [18].

### Molecular genotyping and detection of virulence genes

Multiple molecular typing methods including MRSA identification, SCC*mec* typing of MRSA strains, MLST typing (to determine ST), and *spa* typing were performed as described in a previous study conducted by our team [9].

Thirty-six common virulence genes of *S. aureus* were detected by PCR amplification, including 11 adhesion-associated genes (*bbp*, *clfA*, *clfB*, *cna*, *ebps*, *eno*, *fib*, *fnbA*, *fnbB*, *icaA*, and *icaD*), 12 enterotoxin genes (*sea*, *seb*, *sec*, *sed*, *see*, *seg*, *seh*, *sei*, *sej*, *sem*, *sen*, and *seo*), and 13 other virulence genes (*hla*, *hlb*, *hld*, *hlg*, *hlgv*, *lukM*, *lukED*, *pvl*, *psm*α, *tst*, *eta*, *etb*, and *edin*). All the PCR experiments had been finished in our previously published article [9].

### Antimicrobial susceptibility data

Antimicrobial susceptibility results containing 15 antimicrobials were acquired from the clinical laboratory database of Southwest Hospital. Tested antimicrobials included ciprofloxacin (CIP), clindamycin (CLI), erythromycin (ERY), gentamicin (GEN), linezolid (LNZ), levofloxacin (LVX), moxifloxacin (MFX), nitrofurantoin (NIT), oxacillin (OXA), penicillin (PEN), rifampicin (RIF), trimethoprim/sulfamethoxazole (SXT), tetracycline (TCY), tigecycline (TGC), and vancomycin (VAN). MIC criteria of Clinical and Laboratory Standards Institute (CLSI) were used to determine antimicrobial resistance.

### Other clinical and laboratory data

Basic demographic data including age, sex were anonymously collected. Indicators usually used for evaluating the inflammatory responses of orthopedic patients, including peak values of C-reactive protein (CRP), erythrocyte sedimentation rate (ESR), white blood cell count (WBC), and absolute neutrophil count (ANC) values [9, 15], were collected from all the enrolled patients before an operation intervention and near the bacterial sampling time (because of non-specifically and extremely high values, CRP values of patients with basic inflammatory diseases or recent trauma history were excluded).

### Statistical analysis

IBM SPSS Statistics 23.0 (IBM, Chicago, IL, USA) was used for statistical analysis. In majority of previous studies, categorical variables representing different levels of biofilm-forming capacities were usually used [19, 20]. But the biofilm production in the same level could be very different, so in this study, we used numerical variables (OD_492_ values) for statistical analyses to improve the reliability of this study [21]. Mann–Whitney *U* test or Kruskal–Wallis *H* test was used when comparing the biofilm production between two groups or among three or more groups, respectively. Pearson correlation or spearman rank correlation test was used for correlation analysis. All statistical tests were two-sided and P < 0.05 was considered statistically significant.

## Results

### An overview of biofilm production for studied orthopedic isolates

First of all, to get an overview of biofilm production for all enrolled isolates, the biofilm-forming capacities of all strains were graded. As shown in Table 1, 137 orthopedic *S. aureus* isolates all formed biofilms at various degrees. Among them, 13.1% (18/137), 26.3% (36/137) and 60.6% (83/137) strains presented weak, moderate and strong biofilm production, respectively. More than one-fifth of the strains showed very strong biofilm-forming capacity, with the OD_492_ value > 16ODc (1.175). In addition, no significant difference of biofilm production was found in any group between different genders, or among different sample sources and different ages (Additional file 1: Table S1). The distribution of OD_492_ values between OM and non-OM groups showed no significant difference either (*P* = 0.946, Table 1). These results provided the basis for analyzing the relationship between biofilm production and molecular types among all the orthopedic isolates.

**Table 1:** An overview of biofilm production of studied orthopedic strains

Biofilm formation	Total (n = 137)	OM (n = 60)	Non-OM (n = 77)
Weak (OD ≤ 2ODc), n (%)	18 (13.1)	9 (15.0)	9 (11.7)
Moderate (2ODc < OD ≤ 4ODc), n (%)	36 (26.3)	14 (23.3)	22 (28.6)
Strong (4ODc < OD ≤ 8ODc), n (%)	36 (26.3)	16 (26.7)	20 (26.0)
Strong (8ODc < OD ≤ 12ODc), n (%)	9 (6.6)	3 (5.0)	6 (7.8)
Strong (12ODc < OD ≤ 16ODc), n (%)	9 (6.6)	6 (10.0)	3 (3.9)
Strong (OD > 16ODc), n (%)	29 (21.2)	12 (20.0)	17 (22.1)
OM vs. non-OM	*P*^a^ = 0.946		

^a^By Mann–Whitney U test, indicating no significant difference in biofilm formation between OM and non-OM isolates. ODc = 0.073

### Relationship between biofilm production and MLST or *spa* type

**Fig. 1:**
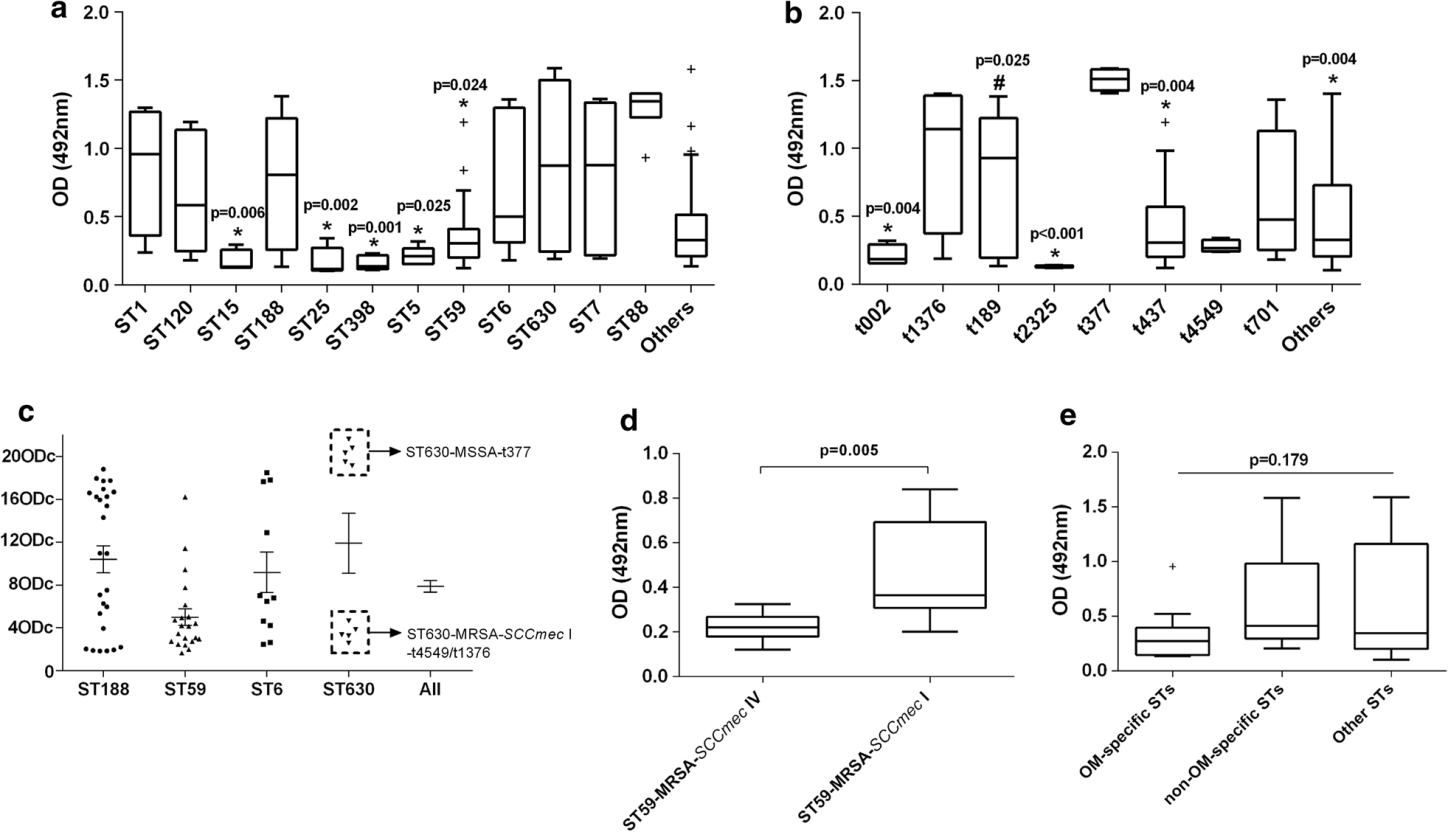
Relationship between biofilm production and molecular genotypes. **a** Box and whisker plot showing the relationship between biofilm production and MLST types. **b** Box and whisker plot showing the relationship between biofilm production and *spa* types. **c** OD_492_ values (indicated by multiples of ODc) distributions of predominant STs (n ≥ 10). **d** Comparison of OD_492_ values between SCC*mec* I and SCC*mec* IV ST59-MRSA strains. **e** Comparison of OD_492_ values among OM-specific STs, non-OM-specific STs and other STs. “*” denotes statistically significant difference. “+” denotes outliers

To determine whether some specific MLST or *spa* types could produce more biofilms, MLST and *spa* typing results of the 137 isolates were summarized in Additional file 2: Table S2. Totally, 34 MLST types and 54 *spa* types were detected (24 MLST types and 36 *spa* types in OM group, 26 MLST types and 35 *spa* types in non-OM group), indicating diverse sources of the orthopedic *S. aureus* infection strains. Biofilm production was compared among various genotypes, and significant differences were found among different MLST and *spa* types (*P *< 0.001, Fig. 1a, b). Specifically, we found that ST88 strains produced significantly stronger biofilms than ST15 (*P *= 0.006), ST25 (*P *= 0.002), ST398 (*P *= 0.001), ST5 (*P *= 0.025) and ST59 (*P *= 0.024) strains (Fig. 1a). As for *spa* types, biofilms produced by t377 strains were significantly stronger than those of t002 (*P *= 0.004), t2325 (*P *< 0.001), t437 (*P *= 0.004), and other *spa* types (*P *= 0.004). In addition, t189 strains were more likely to produce stronger biofilms than t2335 strains (*P *= 0.025) (Fig. 1b).

Next, we wondered if the predominant STs (n ≥ 10) in this study showed some special features when producing biofilms. The biofilm production of predominant STs was summarized in Fig. 1c. We noticed that among ST630 strains, ST630-MSSA-t377 strains all presented OD_490_ values of more than 18 × ODc, while others (ST630-MRSA-SCC*mec*I-t4549/t1376) produced much weaker biolfims (Fig. 1c). ST59-MRSA-SCC*mec*I strains produced significantly stronger biofilms than ST59-MRSA-SCC*mec*IV strains (*P *= 0.005) (Fig. 1c, d). No clue was found to explain the different biofilm production among ST188 or ST6 strains. We also compared the biofilm formation among OM-specific STs, non-OM-specific STs and other STs, and no significant difference was found (*P *= 0.179, Fig. 1e).

### Relationship between biofilm production and carriage of the specific virulence gene

In previous studies, it is controversial on the issue of whether virulence genes could be regarded as indicators for biofilm-forming capacity of *S. aureus* strains [11, 12, 19, 22, 23]. To investigate this question among orthopedic *S. aureus* strains, the carriage of various virulence genes was tested and summarized in Table 2. The biofilm production was compared between specific gene-positive and gene-negative strains. Genes significantly associated with biofilm production were further subjected to spearman correlation analyses (Table 3).

**Table 2:** Comparison of biofilm production between specific gene-positive and gene-negative strains in each group

Strain trait	Total (n = 137)	OM (n = 60)	Non-OM (n = 77)
Adhesion-associated genes, n (%), *P* value^a^			
* bbp* (+)	12 (8.8), 0.670	10 (16.7), 0.330	2 (2.6), 0.259
* cna* (+)	47 (34.3), 0.223	21 (35.0), 0.670	26 (33.8), *0.036*
* ebps* (+)	40 (29.2), 0.558	24 (40.0), 0.214	16 (20.8), 0.552
* fib* (+)	124 (90.5), *0.006*	53 (88.3), 0.120	71 (92.2), *0.014*
* fnbA* (+)	25 (18.2), 0.737	8 (13.3), 0.433	17 (22.1), 0.810
* fnbB* (+)	36 (26.3), 0.401	14 (23.3), 0.832	22 (28.6), 0.182
* icaA* (+)	136 (99.3), -	59 (98.3), -	77 (100.0), -
Enterotoxin genes, n (%), *P* value^a^			
* sea* (+)	11 (8.0), 0.896	7 (11.7), 0.667	4 (5.2), 0.680
* seb* (+)	27 (19.7), 0.234	13 (21.7), 0.634	14 (18.2), *0.032*
* sec* (+)	16 (11.7), 0.104	8 (13.3), 0.083	8 (10.4), 0.558
* sed* (+)	19 (13.9), 0.052	4 (6.7), 0.785	15 (19.5), *0.015*
* see* (+)	2 (1.5), 0.892	0 (0), -	2 (2.6), 0.860
* seg* (+)	8 (5.8), 0.677	5 (8.3), 0.566	3 (3.9), 0.159
* seh* (+)	4 (2.9), 0.328	0 (0), -	4 (5.2), 0.342
* sei* (+)	26 (19.0), *0.004*	14 (23.3), *0.002*	12 (15.6), 0.311
* sej* (+)	9 (6.6), 0.237	4 (6.7), 0.657	5 (6.5), 0.250
* sem* (+)	29 (21.2), *0.009*	15 (25.0), *0.039*	14 (18.2), 0.112
* sen* (+)	21 (15.3), 0.066	9 (15.0), 0.356	12 (15.6), 0.121
* seo* (+)	32 (23.4), *0.002*	19 (31.7), *0.005*	13 (16.9), 0.123
Other toxin genes, n (%), *P* value^a^			
* hla* (+)	132 (96.4), 0.074	55 (91.7), 0.073	77 (100.0), -
* hlb* (+)	45 (32.8), 0.194	18 (30.0), 0.363	27 (35.1), 0.446
* hlg* (+)	127 (92.7), 0.640	58 (96.7), 0.916	69 (89.6), 0.596
* hlgv* (+)	127 (92.7), *0.005*	55 (91.7), 0.073	72 (93.5), *0.035*
* lukED* (+)	90 (65.7), **< ***0.001*	43 (71.7), 0.364	47 (61.0), **< ***0.001*
* pvl* (+)	26 (19.0), 0.319	17 (28.3), 0.589	9 (11.7), 0.419
* edin* (+)	8 (5.8), 0.883	4 (6.7), 0.513	4 (5.2), 0.393
* eta* (+)	5 (3.6), 0.436	2 (3.3), 0.386	3 (3.9), *0.016*
* tst* (+)	5 (3.6), 0.821	3 (5.0), 0.847	2 (2.6), 0.863

Significant differences are in italics

^a^By Mann–Whitney U test, comparing biofilm production (OD_492_ values) between specific gene-positive and gene-negative strains in each group. Genes with carriage rate of 100% (*eno*,*clfA*,*clfB*,*icaD*,*hld* and *psm*α) or 0% (*lukM* and *etb*) were not listed

**Table 3:** Spearman’s correlation analyses between biofilm production and the carriage of specific virulence gene

Strain trait	Spearman’s rho and *P* values		
	Total	OM	Non-OM
*cna* (+)	*P* > 0.05	*P* > 0.05	rho = 0.250, *P *= 0.028
*fib* (+)	rho = 0.234, *P *= 0.006	*P* > 0.05	rho = 0.274, *P *= 0.016
*seb* (+)	*P* > 0.05	*P* > 0.05	rho = −0.245, *P *= 0.031
*sed* (+)	*P* > 0.05	*P* > 0.05	rho = −0.262, *P *= 0.001
*sei* (+)	rho = −0.246, *P *= 0.004	rho = −0.387, *P *= 0.002	*P* > 0.05
*sem* (+)	rho = −0.224, *P *= 0.008	rho = −0.268, *P *= 0.039	*P* > 0.05
*seo* (+)	rho = −0.263, *P *= 0.002	rho = −0.360, *P *= 0.005	*P* > 0.05
*hlgv* (+)	rho = 0.235, *P *= 0.006	*P* > 0.05	rho = 0.240, *P *= 0.036
*lukED* (+)	rho = 0.283, *P *= 0.001	*P* > 0.05	rho = 0.421, *P *< 0.001
*eta* (+)	*P* > 0.05	*P* > 0.05	rho = 0.263, *P *= 0.021

For all enrolled strains, *fib*, *hlgv* and *lukED* genes were positively correlated with biofilm production, while *sei*, *sem* and *seo* genes were negatively correlated. Interestingly, the biofilm production-associated genes were completely different for OM and non-OM strains. In OM group, no adhesion-associated gene was found to be correlated with biofilm production, and only three enterotoxin genes (*sei*, *sem* and *seo*) were found to be negatively correlated. Whereas in non-OM group, genes including *cna*, *fib*, *hlgv*, *lukED* and *eta* were positively correlated with biofilm production, and another two enterotoxin genes, *seb* and *sed*, were negatively correlated with biofilm production (Tables 2 and 3).

**Fig. 2:**
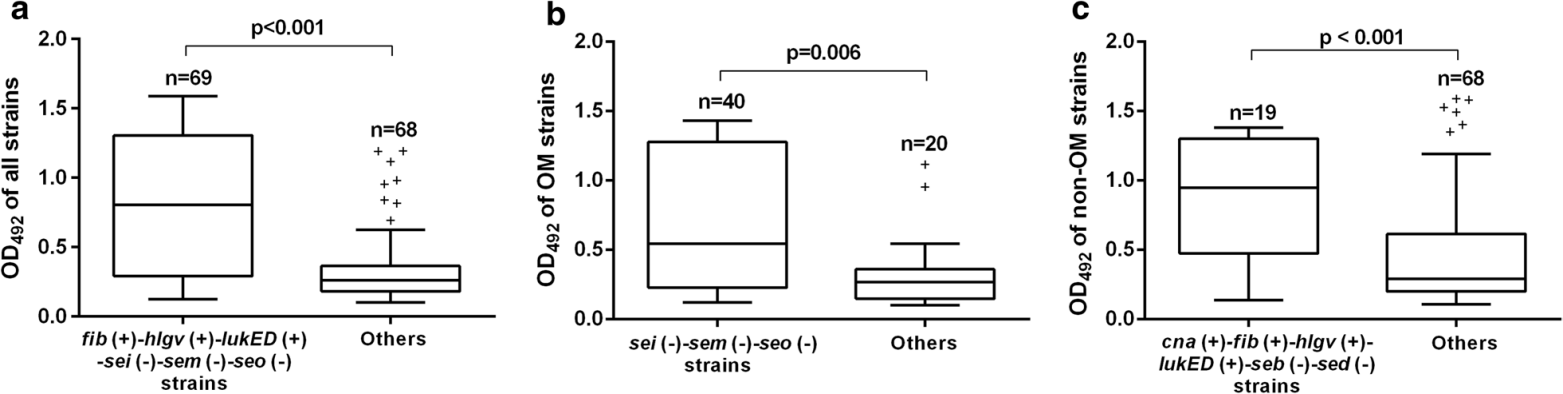
Virulence gene profiles of strains with stronger biofilm production in each group (Box & whisker plot). **a** For all studied orthopedic strains. **b** For OM strains. **c** For non-OM strains. “+” denotes outliers

Based on the above results, we further concluded that strains with the following virulence gene profiles presented significantly stronger biofilm production: 1) *fib* (+)-*hlgv* (+)-*lukED* (+)-*sei* (-)-*sem* (-)-*seo* (-) strains for all the orthopedic isolates (*P *< 0.001); 2) *sei* (-)-*sem* (-)-*seo* (-) strains for OM isolates (*P *= 0.006); 3) *cna* (+)-*fib* (+)-*hlgv* (+)-*lukED* (+)-*seb*(-)-*sed* (-) strains for non-OM isolates (*P *< 0.001) (Fig. 2).

### Relationship between biofilm production and drug resistance

In clinical treatment of *S. aureus*-associated orthopedic infections, biofilm formation and drug-resistance make bacterial eradication more difficult, so we wonder if drug-resistance of *S. aureus* strains was associated with the biofilm formation ability [5, 19]. As summarized in Table 4, for all enrolled strains and non-OM strains, MRSA strains were found to produce significantly weaker biofilms. Weaker biofilms were also observed in the tetracycline-resistant strains due to a positive correlation between MRSA and tetracycline-resistant strains (Additional file 3: Table S3). No correlation was observed between biofilm production and any other kind of drug resistance in any group (Table 4).

**Table 4:** Relationship between biofilm production and drug resistance

Strain trait	Total (n = 137)	OM (n = 60)	Non-OM (n = 77)
	N (%), *P* value^a^		
MRSA	30 (21.9), *0.038* (rho* =−0.169, P = 0.048*)^b^	9 (15.0), 0.553	21 (27.3), *0.042* (*rho = −0.238, P = 0.037*)^b^
MDR	40 (29.2), 0.089	18 (30.0), 0.113	22 (28.6), 0.387
CIP-resistant	12 (8.8), 0.533	6 (10.0), 0.641	6 (7.8), 0.191
CLI-resistant	62 (45.3), 0.151	27 (45.0), 0.202	35 (45.5), 0.417
ERY-resistant	67 (48.9), 0.245	27 (45.0), 0.390	40 (51.9), 0.454
GEN-resistant	12 (8.8), 0.455	6 (10.0), 0.711	6 (7.8), 0.133
LVX-resistant	10 (7.3), 0.260	4 (6.7), 0.598	6 (7.8), 0.368
MFX-resistant	9 (6.6), 0.528	4 (6.7), 0.876	5 (6.5), 0.416
OXA-resistant	32 (23.4), 0.456	13 (21.7), 0.395	19 (24.7), 0.776
PEN-resistant	125 (91.2), 0.888	53 (88.3), 0.340	72 (93.5), 0.367
RIF-resistant	7 (5.1), 0.121	5 (8.3), 0.256	2 (2.6), 0.836
SXT-resistant	13 (9.5), 0.946	4 (6.7), 0.109	9 (11.7), 0.318
TCY-resistant	38 (27.7), *0.031* (*rho = −0.185, P = 0.031*)^b^	21 (35.0), 0.098	17 (22.1), 0.133

All the strains were susceptible to LNZ, TGC and VAN. Significant differences are in italics

^a^By Mann–Whitney U test, comparing biofilm production (OD_492_ values) between resistant and non-resistant strains in each group

^b^Significant factors were further subjected to spearman correlation analyses

### Relationship between biofilm production and patients’ inflammatory responses

It was reported in vitro and in the mouse model that bacterial biofilms could reduce the levels of inflammatory responses to keep the bacteria alive and simultaneously facilitate persistent infections [24, 25]. To explore if this would happen to clinical orthopedic patients, we analyzed the relationship between levels of inflammatory response indicators (including CRP, ESR, WBC and ANC) and the biofilm production of corresponding strains. As shown in Table 5, only for OM patients, CRP and ESR values were negatively correlated with the biofilm production of corresponding *S. aureus* strains. No correlation was detected between biofilm production and WBC or ANC value in any group.

**Table 5:** Pearson’s correlation analyses between biofilm formation and laboratory data

	Total (mean ± SD, rho, *P*)^a^	OM (mean ± SD, rho, *P*)^a^	Non-OM (mean ± SD, rho, *P*)^a^
CRP value	49.4 ± 80.2 (n = 109), −0.098, 0.312	34.6 ± 71.8 (n = 52), *−0.294, 0.034*	62.8 ± 85.0 (n = 57), 0.082, 0.545
ESR value	38.9 ± 31.7 (n = 110), −0.113, 0.240	30.2 ± 29.5 (n = 54), *−0.287, 0.035*	47.2 ± 31.6 (n = 56), 0.098, 0.475
WBC value	8.6 ± 4.3 (n = 118), −0.031, 0.743	8.8 ± 4.7 (n = 57), −0.041, 0.764	8.4 ± 3.9 (n = 61), −0.025, 0.846
ANC value	6.3 ± 4.2 (n = 118), 0.002, 0.987	6.3 ± 4.6 (n = 57), −0.013, 0.926	6.2 ± 3.9 (n = 61), 0.018, 0.891

Significant differences are in italics

^a^Pearson’s correlation analyses between laboratory data and biofilm formation (OD_492_)

## Discussion

Drug resistance is a great concern in the treatment of infectious diseases [3], and biofilm plays an important role in causing drug resistance [8, 10, 20]. *S. aureus* is usually the most common pathogen in orthopedic infections, and skilled in biofilm formation [2, 8]. Hence, investigating the relationship between genetic or phenotypic characteristics and biofilm production among orthopedic *S. aureus* strains, especially for OM-infection strains, is of great significance for clinicians to understand, evaluate and manage biofilm-associated orthopedic infections.

Until now, the issue of whether the molecular type of *S. aureus* is associated with biofilm production is still controversial and lack of investigation. To our knowledge, this study is the first one that investigated the relationship between biofilm-forming capacities and molecular types among orthopedic *S. aureus* strains. According to the results from other specimen resources, some researchers reported that different *spa* types, but not MLST types, could present different biofilm production capacities [20, 26, 27]. However, in several other studies, MLST types were also found to be associated with biofilm-forming capacities. For example, Croes S et al. and Luther MK et al. suggested that MLST CC8 *S. aureus* produced more biofilms than various other MLST types [20, 28]. In this study, we first proposed that ST88, t377 and ST630-MSSA-t377 strains were associated with very strong biofilm producers (Fig. 1a–c). ST88 *S. aureus* is a relatively common clone in Africa, but only sporadic infections were reported in China [29]. No research focusing on biofilm-forming capacity of ST88 *S. aureus* was found. However, it should be noted that a considerable proportion of ST88 *S. aureus* strains carried *pvl* gene [29], a pore-forming toxin gene that plays a crucial role in the pathogenicity of *S. aureus* [9]. Five of the six t377 strains in this study belonged to ST630-MSSA, ST630-MSSA-t377 strains presented as sporadic infections or relatively dominant strains in China [30, 31], but the strong biofilm-forming capacity had not been realized in previous studies. Our results suggest that special attention should be paid to the biofilm infections caused by ST88 and ST630-MSSA-t377 strains, and larger epidemiological investigations are needed to verify their very strong biofilm-forming capacity and to explore the underlying mechanisms. For ST59, one of the most prevalent MRSA clones in Asia, although Yang X et al. reported that ST59-SCC*mec*IV strains isolated from Chinese children were more likely to form strong biofilm [21], our results showed that ST59-MRSA-SCC*mec*I produced significantly stronger biofilms than ST59-MRSA-SCC*mec*IV strains (Fig. 1d), suggesting that different environments or specimen sources might also affect the biofilm-forming capacity.

Exploring the relationship between biofilm production and carriage of virulence genes may provide biomarkers for diagnosis and targets for biofilm eradication. Although Tang J et al. indicated that a single gene or subset of genes cannot be utilized as an indicator of biofilm production [23], In some other studies, traditional adhesion-associated genes including *ebps*, *ica* and *fnbA* were suggested to be positively associated with biofilm production [11, 22]. Exotoxin gene *lip* and *hla* genes were also verified to contribute to biofilm formation [12, 32]. Different from the above results, in this study, we first found that the *fib*, *hlgv* and *lukED* gene was positively correlated, while *sei*, *sem* and *seo* genes were negatively correlated with biofilm production of orthopedic *S. aureus* strains (Tables 2, and 3). Interestingly, we noticed that genes correlated with biofilm production were completely different for OM and non-OM strains (Table 3). This result implies that different factors or mechanisms participate in the biofilm production of OM and non-OM strains. Also worth noting is that, in each group, we proposed a virulence gene profile with which the strains tended to produce stronger biofilm (Fig. 2). These profiles probably reflect some features of strong biofilm producers.

It has been implied in many kinds of bacteria that antimicrobial susceptibility could be related to biofilm production [33–35]. But the exact relationship between them is still confusing. For *S. aurues*, several studies suggested that biofilm-forming strains were more likely to be MDR, and MRSA produced stronger biofilm than MSSA strains [13, 21]. However, in some other studies, no correlation between drug resistance and biofilm-forming capacity was found [36]. In this study, only a negative correlation was found between MRSA and biofilm production among non-OM strains (Table 3). So we speculate that a single antimicrobial resistance is unable to alter the biofilm-forming capacity, but acquisition of the *mecA* gene, which mediates the transition from MSSA to MRSA [14], may change the process of biofilm-forming of orthopedic non-OM strains by unknown mechanisms.

To realize persistent infections (such as persistent OM), bacteria usually need to adjust themselves to a state of low-level inflammatory responses, and biofilm formation is an important way to achieve this [24]. Although this phenomenon has been observed in vitro and in the mouse model [24, 25], and Klingenberg C et al. also found that there was an association between a lower CRP value and biofilm-positive isolates in coagulase-negative staphylococci [36], it has not been verified in clinical patients with *S. aureus* orthopedic infections. Here, we found that, among OM patients, CRP and ESR values were negatively correlated with biofilm production (Table 5). This result further implies that, by reducing CRP and ESR values, bioflim plays a significant role in the course of persistent OM infections, and the two data could be potential indicators for biofilm-associated *S. aureus* OM infections.

## Conclusions

In summary, we characterized the relationship between biofilm production and various genetic and phenotypic characteristics of orthopedic *S. aureus* strains isolated from OM and non-OM infections. Some MLST and *spa* types were shown to be associated with biofilm production. Strains with specific virulence gene profiles could be more likely to be strong biofilm producers, and this profile was completely different for OM and non-OM isolates. Not any single drug resistance was found to be associated with biofilm production. At last, we observed among OM patients that stronger biofilm producers tended to lead to lower inflammatory responses. The results of this study may help clinicians to better understand, evaluate and manage biofilm-associated orthopedic infections and provide potential targets for biofilm clearance.

## Supplementary information

**Additional file 1: Table S1.** OD_492_ distribution between different genders, or among different sample sources, different ages in each group.

**Additional file 2: Table S2.** Molecular typing results of each group.

**Additional file 3: Table S3.** Correlation analyses between MRSA and TCY-resistance by Kendall’s tau test.

## Data Availability

All the dataset of this article is available from the corresponding author if reasonably requested.
