# Supplementary material for: Investigation of biofilm production and its association with genetic and phenotypic characteristics of OM (osteomyelitis) and non-OM orthopedic Staphylococcus aureus
Source: Ann Clin Microbiol Antimicrob. 2020 Mar 26;19:10. doi: 10.1186/s12941-020-00352-4 (PMC7099788; doi:10.1186/s12941-020-00352-4)
Supplement: Supplementary file 1 — Additional file 1: Table S1. OD492 distribution between different genders, or among different sample sources, different ages in each group. [file 12941_2020_352_MOESM1_ESM.docx]

**Table S1.** OD_492_ distribution between different genders, or among different sample sources, different ages in each group

| Grouping method | *P* values by non-parametric test | | |
| --- | --- | --- | --- |
|  | Total (n=137) | OM (n=60) | non-OM (n=77) |
| Sample source^1^ | 0.668 | 0.629 | 0.159 |
| Sex^2^ | 0.656 | 0.079 | 0.965 |
| Age group^3^ | 0.421 | 0.099 | 0.439 |

^1^By Kruskal–Wallis test, sample sources include blood, marrow, joint fluid, pus, wound, and tissue around the infection site. ^2^By Mann–Whitney U test, including male and female groups. ^3^By Kruskal–Wallis test, including four age groups, ≤ 30, ≤40, ≤50 and >50 years old.
