# Supplementary material for: Investigation of biofilm production and its association with genetic and phenotypic characteristics of OM (osteomyelitis) and non-OM orthopedic Staphylococcus aureus
Source: Ann Clin Microbiol Antimicrob. 2020 Mar 26;19:10. doi: 10.1186/s12941-020-00352-4 (PMC7099788; doi:10.1186/s12941-020-00352-4)
Supplement: Supplementary file 2 — Additional file 2: Table S2. Molecular typing results of each group. [file 12941_2020_352_MOESM2_ESM.docx]

**Table S2.** Molecular typing results of each group

| Total (n=137) | | OM (n=60) | | non-OM (n=77) | |
| --- | --- | --- | --- | --- | --- |
| MLST (n) | *spa* type (n) | MLST (n) | *spa* type (n) | MLST (n) | *spa* type (n) |
| ST188 (26) | t189 (23), t2883 (1),  t8914 (1),16342 (1) | ST188 (11) | T189 (10), t16342 (1) | ST188 (15) | T189 (13), t2883 (1), t8914 (1) |
| ST59 (21) | t437 (17), t441 (3), t8940 (1) | ST59 (9) | t437 (8), t441 (1) | ST59 (12) | t437 (9), t441 (2) , t8940 (1) |
| ST6 (11) | t701 (9), t304 (1), t9476 (1) | ST6 (5) | t701 (4), t304 (1) | ST6 (6) | t701 (5), t9476 (1) |
| ST630 (10) | t377 (5), t4549 (4), t1376 (1) | ST630 (2) | t377 (2) | ST630 (8) | t377 (3), t4549 (4), t1376 (1) |
| ST120 (6) | t159 (2), t7290 (2),  t2019 (1), t16519 (1) | ST120 (4) | t159 (1), t7290 (1),  t2019 (1), t16519 (1) | ST120 (2) | t159 (1), t7290 (1) |
| ST88 (6) | t1376 (3),t2592 (1),  t13862 (1), t16520 (1) | ST88 (4) | t1376 (1),t2592 (1),  t13862 (1), t16520 (1) | ST88 (2) | t1376 (2) |
| ST25 (5) | t078 (1), t081 (1), t401 (1),  t1521(1), t7642 (1) | ST25 (3) | t081 (1), t1521(1), t7642 (1) | ST25 (2) | t078 (1), t401 (1) |
| ST398 (5) | t034 (2), t571 (1),  t1451 (1), t2325 (1) | ST398 (1) | t2325 (1) | ST398 (4) | t034 (2), t571 (1), t1451 (1) |
| ST5 (5) | t002 (4), t1791 (1) | ST5 (1) | t002 (1) | ST5 (4) | t002 (3), t1791 (1) |
| ST7 (5) | t2663 (2), t7164 (2), t6193 (1) | ST7 (2) | t2663 (1), t6193 (1) | ST7 (3) | t2663 (1), t7164 (2) |
| ST1 (4) | t116 (1), t127 (2), t286 (1) | ST1 (3) | t116 (1), t127 (1), t286 (1) | ST1 (1) | t127 (1) |
| ST15 (4) | t2325 (3),t6130 (1) | ST15 (1) | t2325 (1) | ST15 (3) | t2325 (2),t6130 (1) |
| ST1821 (2) | t3930(1), t16521 (1) | ST1821 (1) | t3930(1) | ST1821 (1) | t16521 (1) |
| ST573 (2) | t1839 (1), t10629 (1) | ST573 (1) | t1839 (1) | ST573 (1) | t10629 (1) |
| ST72 (2) | t148 (2) | ST72 (1) | t148 (1) | ST72 (1) | t148 (1) |
| ST8 (2) | t9101 (2) | ST8 (1) | t9101 (1) | ST8 (1) | t9101 (1) |
| ST121 (2) | t2019(1), t2091 (1) | ST121 (2) | t2019(1), t2091 (1) |  |  |
| ST30 (2) | t021 (2) | ST30 (2) | t021 (2) |  |  |
| ST338 (2) | t437 (2) |  |  | ST338 (2) | t437 (2) |
| ST1301 (1) | t3666 (1) | ST1301 (1) | t3666 (1) |  |  |
| ST2315 (1) | t11687 (1) | ST2315 (1) | t11687 (1) |  |  |
| ST3538 (1) | t8940 (1) | ST3538 (1) | t8940 (1) |  |  |
| ST45 (1) | t116 (1) | ST45 (1) | t116 (1) |  |  |
| ST509 (1) | t375 (1) | ST509 (1) | t375 (1) |  |  |
| ST702 (1) | t3092 (1) | ST702 (1) | t3092 (1) |  |  |
| ST1409 (1) | t062 (1) |  |  | ST1409 (1) | t062 (1) |
| ST149 (1) | t45 (1) |  |  | ST149 (1) | t45 (1) |
| ST2139 (1) | t189 (1) |  |  | ST2139 (1) | t189 (1) |
| ST22 (1) | t11413 (1) |  |  | ST22 (1) | t11413 (1) |
| ST2235 (1) | t377 (1) |  |  | ST2235 (1) | t377 (1) |
| ST239 (1) | t030 (1) |  |  | ST239 (1) | t030 (1) |
| ST2592 (1) | t127 (1) |  |  | ST2592 (1) | t127 (1) |
| ST3539 (1) | t7642 (1) |  |  | ST3539 (1) | t7642 (1) |
| ST9 (1) | t899 (1) |  |  | ST9 (1) | t899 (1) |
