# Supplementary material for: Investigation of biofilm production and its association with genetic and phenotypic characteristics of OM (osteomyelitis) and non-OM orthopedic Staphylococcus aureus
Source: Ann Clin Microbiol Antimicrob. 2020 Mar 26;19:10. doi: 10.1186/s12941-020-00352-4 (PMC7099788; doi:10.1186/s12941-020-00352-4)
Supplement: Supplementary file 3 — Additional file 3: Table S3. Correlation analyses between MRSA and TCY-resistance by Kendall’s tau test. [file 12941_2020_352_MOESM3_ESM.docx]

**Table S3.** Correlation analyses between MRSA and TCY-resistance by Kendall's tau test

|  | Total (n=137) | OM (n=60) | non-OM (n=77) |
| --- | --- | --- | --- |
| MRSA (n, %) | 30, 21.9% | 9, 15% | 21, 27.3% |
| TCY-resistance (n, %) | 38, 27.7% | 21, 35.0% | 17, 22.1% |
| Kendall's tau | **0.184** | **0.377** | 0.096 |
| P value | **0.031** | **0.004** | 0.403 |

Significant differences are in boldface.
